# Mechanisms and Management of Immune Checkpoint Inhibitor-Related Cardiac Adverse Events

**DOI:** 10.31662/jmaj.2021-0001

**Published:** 2021-04-02

**Authors:** Hiroshi Kadowaki, Hiroshi Akazawa, Junichi Ishida, Issei Komuro

**Affiliations:** 1 Department of Cardiovascular Medicine, Graduate School of Medicine, The University of Tokyo, Tokyo, Japan

**Keywords:** CTLA-4, immune-related adverse events, myocarditis, PD-1, PD-L1

## Abstract

Onco-cardiology recently emerged as a novel discipline to provide effective cardioprotective care against cancer therapeutics-related cardiac adverse events (CAEs) and support the continuity of optimal cancer treatment. Immune checkpoint inhibitors (ICIs) have revolutionized cancer therapy and dramatically improved outcomes in patients with advanced or refractory cancers. However, ICIs intrinsically stimulate systemic immune responses and can potentially induce a spectrum of immune-related adverse events (irAEs), which can affect any organs of the body. The manifestation of cardiac irAEs includes myocarditis, arrhythmias and conduction abnormalities, and pericardial diseases. Takotsubo-like cardiomyopathy is also included as a manifestation of ICI-related CAEs, but the pathophysiological relevance is unclear. Although the incidence is rare, ICI-related CAEs are life-threatening and potentially fatal. Elucidating pathophysiology and establishing management measures of ICI-related CAEs are one of the most urgent challenges in the field of onco-cardiology.

## Introduction

Cancer remains to be the first or second leading cause of death in developed countries, but over the past few decades, recent progress in cancer therapy has remarkably improved the long-term outcome for patients with cancer. At the same time, cardiovascular complications related to chemotherapy and radiotherapy emerge as one of the major factors influencing the prognosis or quality of life. Under such circumstances, “onco-cardiology” or “cardio-oncology” attracts much attention worldwide as a new discipline to provide cardioprotective care for patients with cancer and prevent inappropriate interruption of potentially lifesaving cancer treatment ^(1), (2)^. Especially, in the field of onco-cardiology, managing cardiac adverse events (CAEs) related to immune checkpoint inhibitors (ICIs) is becoming a major concern.

In the early 20th century, it was observed that some substances in the blood from patients with cancer interfered with the destruction of tumor cells by lymphocytes, but the pivotal molecules responsible for this phenomenon have not been identified until recently ^(3)^. In the 1960s-1970s, it became evident that some molecules inactivated human lymphocytes harboring cytotoxic activity on tumor cells. In 1987, the molecule was identified on the surface of T-lymphocyte and named cytotoxic T-lymphocyte antigen 4 (CTLA-4) ^(4)^. Program death 1 (PD-1) was originally identified to participate in programmed cell death by Dr. Tasuku Honjo and his colleagues in 1992 ^(5)^ and was thereafter added to a list of molecules regulating anticancer immune response. The inhibition of these “immune checkpoints” accelerates an active immune response by enhancing priming and activation of T cells and potentiating cytotoxic activity of T cells on their target tumor cells ^(6), (7)^. Dr. James P. Allison firstly reported the inhibitory effects of CTLA-4 on anticancer immune response in mice injected with colon carcinoma cells ^(8)^. In human, a landmark trial demonstrated that ipilimumab, an antibody against CTLA-4, improved overall survival in patients with metastatic melanoma ^(9)^; it was approved by the US Food and Drug Administration (FDA) in 2011 ^(10)^. Nivolumab and pembrolizumab, antibodies against PD-1, were also approved in 2014 by the FDA ^(10)^. These ICIs have dramatically improved mortality in patients with advanced or refractory cancer ^(9), (11)^. Drs. James P. Allison and Tasuku Honjo were awarded the 2018 Nobel Prize in Medicine and Physiology for their accomplishment in cancer immunotherapy ^(12)^. In addition to ipilimumab, nivolumab, and pembrolizumab, cemiplimab, an antibody against PD-1, and avelumab, atezolizumab, and durvalumab, antibodies against program death ligand 1 (PD-L1), are currently approved by the FDA, and the indications of ICIs for cancer therapy have been expanding (Table 1) ^(13)^.

**Table 1.** Immune Checkpoint Inhibitors Approved by the US Food and Drug Administration (FDA).

Immune checkpoint inhibitors	Product name	Target	Approved indication by the FDA	Date of approval
Ipilimumab	Yervoy	CTLA-4	Melanoma, renal cell carcinoma, colorectal cancer	March 2011
Pembrolizumab	Keytruda	PD-1	Melanoma, non-small cell lung cancer, non-squamous cell lung cancer (with high PD-L1 expression), renal cell carcinoma, classic Hodgkin's lymphoma, gastric or gastroesophageal junction adenocarcinoma, urothelial carcinoma, cervical cancer, large B-cell lymphoma, Merkel cell carcinoma	September 2014
Nivolumab	Opdivo	PD-1	Melanoma, non-small cell lung cancer, small cell lung cancer, renal cell carcinoma, Hodgkin’s lymphoma, head and neck squamous cell carcinoma, hepatocarcinoma, colorectal cancer	December 2014
Avelumab	Bavencio	PD-L1	Merkel cell carcinoma, urothelial carcinoma, renal cell carcinoma	November 2015
Atezolizumab	Tecentriq	PD-L1	Urothelial carcinoma, non-small cell lung cancer, breast cancer, non-squamous non-small cell lung cancer, small-cell lung cancer	May 2016
Durvalumab	Imfinzi	PD-L1	Urothelial carcinoma, non-small cell lung cancer	February 2016
Cemiplimab	Libtayo	PD-1	Cutaneous squamous cell carcinoma	September 2018

Although ICIs have revolutionized cancer therapy, the use of ICIs is associated with a spectrum of immune-related adverse events (irAEs), potentially affecting most organs of the body ^(14)^. In particular, ICI-related CAEs are life-threatening and emerge as the most urgent problem in the field of onco-cardiology ^(15), (16)^. In this article, we update current knowledge of the mechanisms and management of ICI-related CAEs.

## Molecular Mechanisms of Anticancer Effect

Anticancer immunity consists of a series of stepwise events characterized by the cyclic coordination of numerous factors, leading to the amplification of T-cell responses ^(7)^. The recognition of cancer cell antigens by dendritic cells initiates the first step of the anticancer immune cycle. These neo-antigens are presented to T cells via major histocompatibility complex (MHC), resulting in priming and activation of effector T-cell to cancer cell (the priming phase). The activity of the immune response is determined at this stage, with a balance of effector T cells versus regulatory T cells (Tregs). Activated effector T cells traffic to and infiltrate tumor bed and injure cancer cells by binding through interaction between T-cell receptor (TCR) and cognate antigen bound to MHC (the effector phase). The killing of cancer cells triggers the release of additional cancer cell antigens, entering into the next cycle to reinforce anticancer activity through the amplification of T-cell immunity to cancer cells ^(7)^.

**Figure 1.**
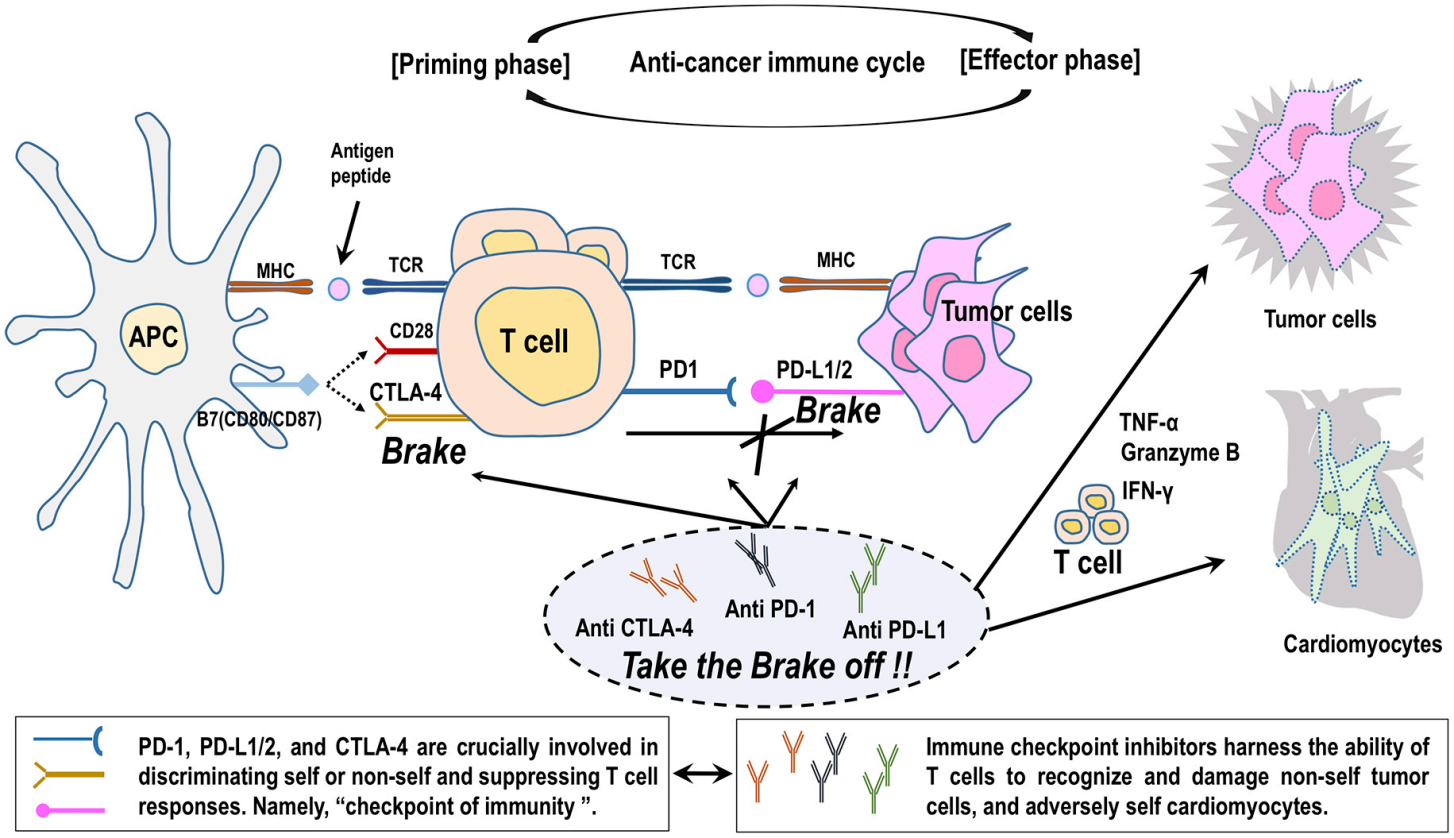
Effects of immune checkpoint inhibitors on tumor cells and cardiomyocytes. Inhibition of suppressive factors called “immune checkpoints” accelerates activated T-cell to invade and injure tumor cells but may also allow the immune system to attack normal organs in our body, including the heart. APC, antigen-presenting cell; CTLA-4, cytotoxic T-lymphocyte antigen 4; MHC, major histocompatibility complex; PD-1, program death ligand 1; PD-L1/PD-L2, programmed cell death ligand 1 or ligand 2; TCR, T-cell receptor; TNF-α, tumor necrosis factor-α; IFN-γ, interferon-γ.

The molecular targets of ICIs, such as CTLA-4 and PD-1/PD-L1, negatively regulate T-cell activity at multiple phases of the anticancer immune cycle. CTLA-4 competes with CD28 on the T-cell for binding to B7-1 (CD80) on the antigen-presenting cells and prevent costimulatory signal from CD28 and B7-1 binding, thereby leading to the suppression of T-cell immunity in the priming phase ^(7)^. CTLA-4 is also expressed in Tregs and is involved in their suppressive function ^(17)^. On the other hand, PD-L1/PD-L2 binds to PD-1 on T cells, resulting in inhibitory checkpoint signaling that leads to T-cell exhaustion and suppression of the immune system against cancer in the effector phase ^(7)^. Cancer cells protect themselves from anticancer immunity by expressing PD-L1/PD-L2 ^(18), (19)^. ICIs, targeting CTLA-4, PD-1, or PD-L1/PD-L2, inhibit these immune checkpoint pathways and thereby boost immunological responses against cancer cells (Figure 1) ^(14), (20)^.

## Pathophysiology of Cardiac irAEs

**Figure 2.**
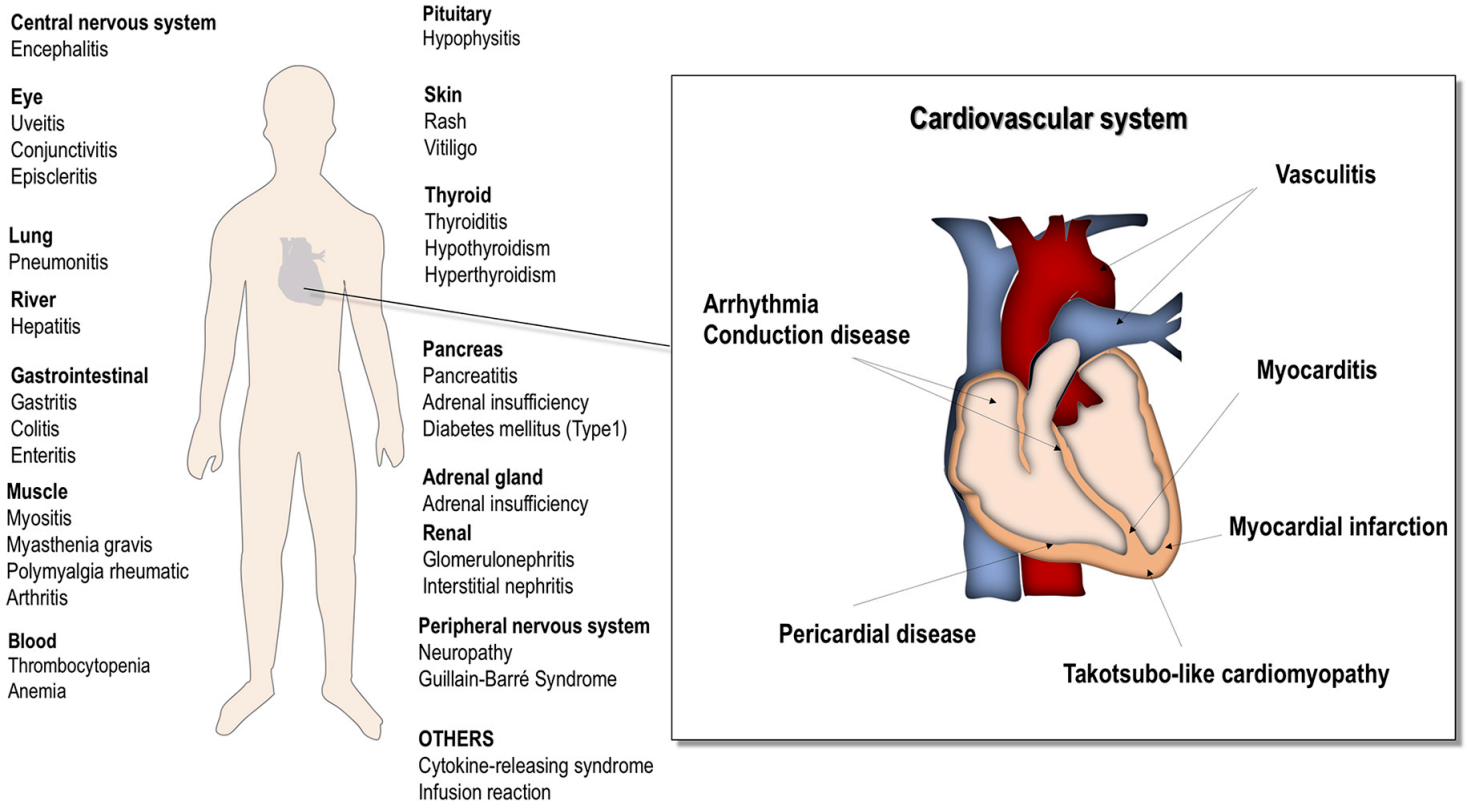
Immune-related adverse events in each organ.

The pathophysiology of ICI-related cardiotoxicity is not fully elucidated ^(21), (22)^. ICIs specifically take the brakes off the T cells attacking cancer cells and adversely arouse excessive immunity against normal organs in our body, leading to irAEs ^(14)^. It is proposed that interference with the immunosuppressive pathway by treatment with ICIs could allow T cells to be activated without control and infiltrate into the heart (Figure 2) ^(14)^. CTLA-4-deficient mice developed lethal myocarditis and pancreatitis with lymphocytic infiltration and tissue destruction and died within 3-4 weeks of age ^(23)^. Anti-CTLA-4 antibody interferes with the interaction between CTLA-4 and B7-1, resulting in lowering the threshold for the activation of cardiac antigen-reactive T cells. In addition, CTLA-4 blockade impairs Tregs-mediated immunologic self-tolerance and activates cardiac antigen-reactive T cells ^(17), (24)^. Similarly, anti-PD-1 and PD-L1 antibodies interfere with mutual interaction and induce the activation of cardiac antigen-reactive T cells. Mice deficient for PD-1 or PD-L1 manifested various phenotypes of autoimmune cardiomyopathy dependently on their genetic backgrounds ^(14)^. In BALB/c mice, PD-1 deficiency caused autoimmune dilated cardiomyopathy without myocardial infiltration of inflammatory cells ^(25)^. Alternatively, there was diffuse and abundant deposition of C3 complement and immunoglobulin reactive to cardiac troponin I ^(25), (26)^. Curiously, there is no report to date demonstrating that autoantibodies are the underlying cause of cardiotoxicity in ICI-treated patients with cancer ^(21), (22)^. Mice deficient for PD-1 or PD-L1 similarly developed fatal myocarditis with massive infiltration of CD4^+^ and CD8^+^ T cells in an autoimmune-prone MRL-*Fas^lpr/lpr^* background ^(27), (28)^, but PD-1-deficient mice did not develop autoimmune cardiomyopathy in a non-autoimmune-prone C57BL/6 background ^(29)^. The upregulation of PD-L1 expression was observed on the surface of injured cardiomyocytes and infiltrating CD8^+^ T cells in the heart of patients with ICI myocarditis, as well as PD-1-deficient MRL-*Fas^lpr/lpr^* mice ^(27), (30), (31)^. An experimental study using a mouse model of cytotoxic T-cell-mediated myocarditis indicated that PD-L1 upregulation by interferon-γ functions to protect cardiomyocytes from inflammatory injuries ^(32)^. Immune checkpoint blockade abrogates these protective responses, leading to the aggravation of inflammatory injury to myocardium ^(21), (22)^.

Next-generation sequencing of TCRs in the heart of two autopsied patients with fulminant ICI myocarditis revealed that CD4^+^ and CD8^+^ T-cell clones in the myocardium were identical to those in tumors ^(30)^. In addition, striated muscle-specific antigens, such as desmin and troponin, were abundantly observed in tumors of these patients, suggesting that the antigens targeted by infiltrating are common or homologous between the myocardium and cancer cells ^(30)^. It is also possible that certain TCRs targeted dissimilar antigens ^(30), (33)^; further assessment of T-cell clonality and identification of the causative antigens in a larger number of cases will provide a clue to the exact mechanism underlying ICI myocarditis.

## Clinical Features and Management of ICI-Related CAEs

Phase 3 trials of ICIs indicated an extremely low incidence of cardiotoxicity ^(34), (35)^, but life-threatening cases of ICI-related cardiac complications have been sporadically reported after their market launch ^(30), (36), (37), (38)^. According to an observational, retrospective, pharmacovigilance study that evaluated ICI-related cardiovascular complications using VigiBase, the global database of individual case safety reports by the World Health Organization (WHO), ICI treatment was associated with higher reporting of myocarditis (0.39% for ICIs; reporting odds ratio [ROR], 11.21; 95% confidence interval [CI], 9.36-13.43), pericardial diseases (0.30% for ICIs; ROR, 3.80; 95% CI, 3.08-4.62), and vasculitis (temporal arteritis and polymyalgia rheumatica) (0.26% for ICIs; ROR, 1.56; 95% CI, 1.25-1.94) (Figure 2) ^(39)^. Another VigiBase analysis revealed that ICI treatment was also associated with higher reporting of Takotsubo cardiomyopathy (stress cardiomyopathy) (0.03% for ICIs; ROR, 3.39; 95% CI, 1.96-5.86) (Figure 2) ^(40)^.

### Myocarditis

ICI myocarditis is a rare but clinically important irAE because it is associated with high mortality ^(15), (16), (33)^. According to the Bristol-Myers Squibb corporate safety databases enrolling 20,594 patients treated with nivolumab and nivolumab plus ipilimumab, myocarditis was reported in 18 patients (0.09%), and patients receiving combination therapy had higher incidence (0.27%) than those receiving nivolumab alone (0.06%) ^(30)^. In a single-center registry (Massachusetts General Hospital, MA) of 964 ICI-treated patients, myocarditis was noted in 11 patients (1.14%) ^(41)^. In a multicenter registry collecting 35 patients with ICI myocarditis, 16 patients (46%) developed major adverse cardiac events (MACEs): cardiovascular death (n = 6), cardiogenic shock (n = 3), cardiac arrest (n = 4), or complete heart block (n = 3) ^(41)^. According to a VigiBase analysis, severe adverse event was documented in the majority (84%) of ICI myocarditis cases, with death occurring in 50% ^(39)^. Another report using the WHO database VigiLyze-VigiBase also revealed that myocarditis presented by far the highest mortality (39.7%) among all organ-specific irAEs ^(42)^. Importantly, ICI myocarditis cases were often complicated with concomitant irAEs affecting other organ systems, such as myositis (25%), myasthenia gravis (11%), pneumonitis or pneumonia (11%), and hepatitis (11%) ^(39)^. A majority of myocarditis developed early after the initiation of ICI therapy. In a multicenter registry collecting 35 patients with ICI myocarditis, the median time to onset from the first ICI therapy was 34 days (interquartile range [IQR], 21-75 days), with 81% presenting within 3 months ^(41)^. A VigiBase analysis also reported that the median time to onset was 30 days (IQR, 18-60 days) ^(39)^.

**Figure 3.**
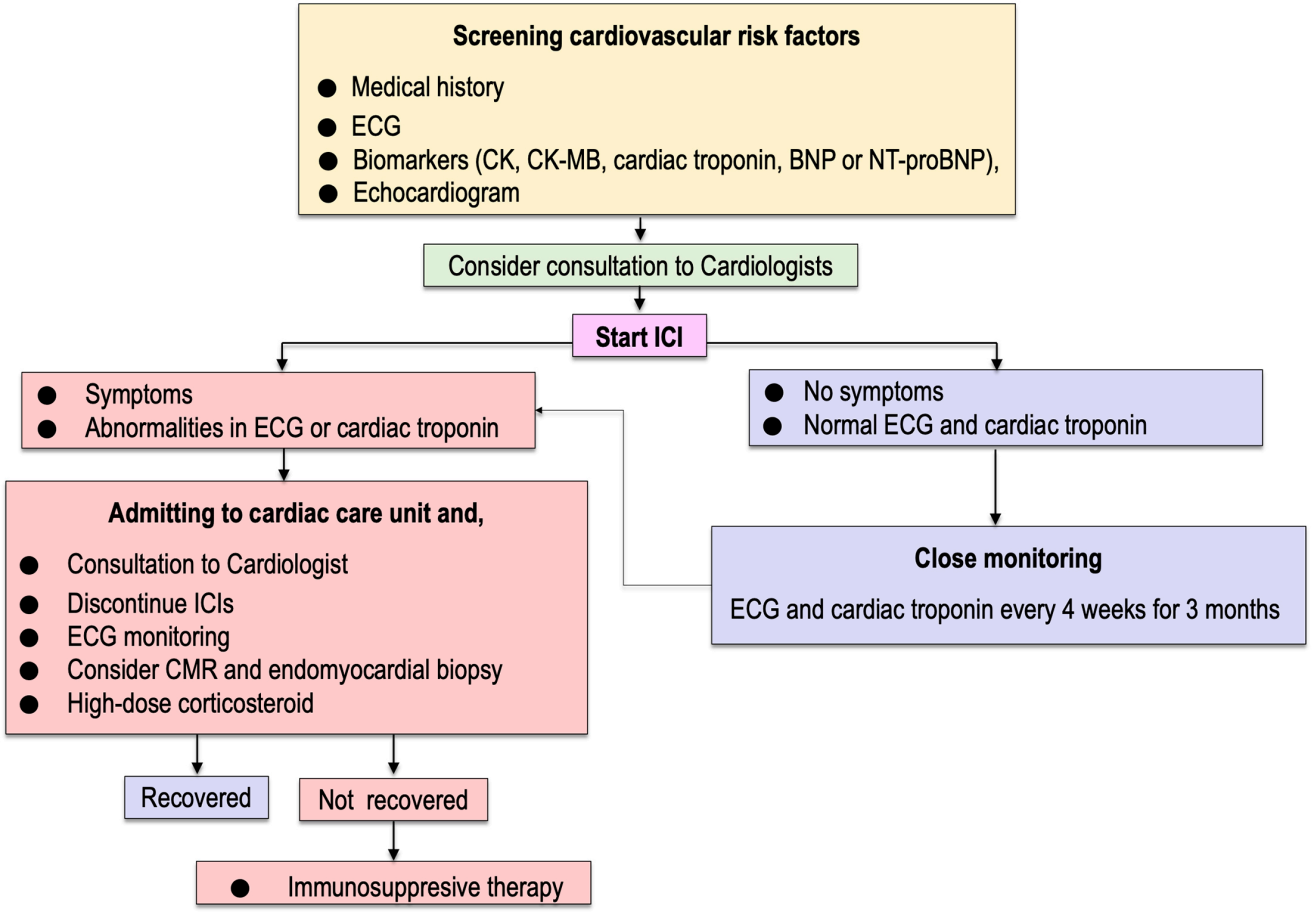
Flow diagram for optimal cardiovascular follow-up of patients treated with ICIs. BNP, B-type natriuretic peptide; CK, creatinine kinase; CK-MB, creatine kinase myocardial band; CMR, cardiac magnetic resonance; ECG, electrocardiogram; NT-proBNP, N-terminal pro BNP; ICIs, immune checkpoint inhibitors.

Patients with ICI myocarditis manifest nonspecific symptoms including dyspnea, chest pain, fatigue, palpitations, syncope, or dizziness, which overlap with common symptoms observed in patients with cancer. Since ICI myocarditis is characterized by rapidly progressive clinical deterioration leading to intractable heart failure or lethal arrhythmias, catching the first signs for early diagnosis of myocarditis is crucial. At the time of presentation, almost all patients with ICI myocarditis showed elevation of cardiac troponin (94%) ^(41)^. Abnormal electrocardiogram (ECG) was also observed in a high proportion of patients (89%), but left ventricular ejection fraction (LVEF) obtained using echocardiography was normal (≥ 50%) in 51% of overall patients and even in 38% of patients who developed MACE ^(41)^. However, ECG assessment should be conducted for diagnosis in all patients who are suspected of CAEs, including myocarditis, pericardial diseases, or Takotsubo-like syndrome ^(33), (43)^. Endomyocardial biopsy is of the highest value for diagnosing ICI myocarditis, but it is invasive and requires technical expertise ^(33), (43)^. Cardiac magnetic resonance (CMR) is a preferred imaging modality to provide evidence of myocardial inflammation by demonstrating myocardial edema and nonischemic myocardial injury ^(33), (43)^. In an international multicenter registry including 103 patients with pathology-proven ICI myocarditis, late gadolinium enhancement (LGE) was present only in 48% of overall patients (55% of patients with reduced LVEF and 43% of patients with preserved EF) ^(44)^. The rate of LGE-positive patients in this study was much lower than that observed for non-ICI myocarditis ^(45)^. The positive rate of LGE increased according to the interval between clinical presentation and CMR examination (21.6% when CMR was performed within 4 days of admission; 72.0% when CMR was performed on day 4 or later) ^(44)^. Similarly, the rate of patients with elevated T2-weighted short tau inversion recovery (STIR) was present only in 28% of overall patients ^(44)^. Therefore, endomyocardial biopsy should be considered in patients with clinical suspicion of ICI myocarditis, even when they have normal LVEF, negative LGE, and T2-weighted STIR in CMR ^(44), (46)^, because the delay in diagnosis and treatment may lead to worse outcomes ^(47)^. We propose an algorithm for cardiovascular surveillance of patients who are treated with ICIs (Figure 3) ^(38)^. Importantly, like other proposed algorithms for the management of ICI myocarditis ^(22), (41), (43)^, the algorithm should be optimized according to medical resource capacities of individual facilities and updated after validation of the effectiveness.

According to the guidelines, interrupting ICI therapy is recommended when cardiac irAEs of any grade are present ^(48), (49)^. The use of corticosteroids is chosen for initial treatment of ICI myocarditis. It was reported that a higher initial dose (i.e., intravenous methylprednisolone, 1,000 mg/day) and early initiation of corticosteroids (i.e., within 24 hours of admission) was associated with a lower rate of MACE in multicenter registries ^(41), (47)^. When patients are refractory or inadequately responsive to corticosteroids, immunosuppression therapy using mycophenolate mofetil, tacrolimus, immunoglobulin, antithymocyte globulin, infliximab, alemtuzumab, and abatacept or plasmapheresis have been considered ^(14), (22), (33), (41), (50), (51)^. The use of infliximab, a monoclonal antibody against tumor necrosis factor (TNF)-α, should be avoided in patients with decreased LVEF or symptomatic heart failure because of the risk of worsening heart failure ^(52)^. More recently, it was reported that the use of abatacept, a CTLA-4 agonist, induced the resolution of corticosteroid-refractory ICI myocarditis ^(51)^. Abatacept may be a promising candidate as an effective drug for reversing immune checkpoint inhibition in patients with corticosteroid-refractory myocarditis caused by both anti-CTLA-4 and anti-PD-1/PD-L1 antibodies ^(33), (51)^. The number of cases with successful treatment by immunosuppression therapy is currently limited, and further accumulation of evidence is needed for the risk-benefit consensus on immunosuppression therapy other than corticosteroids.

### Arrhythmia and conduction abnormalities

Arrhythmias and conduction abnormalities are commonly observed in patients with ICI-related CAEs ^(15), (16)^. In a pooled analysis of 30 patients with ICI-related complications, atrial fibrillation, ventricular arrhythmia, and conduction disorders were present in 9 patients (30%), 8 patients (27%), and 5 patients (17%), respectively ^(53)^. Immediate intervention, such as cardioversion and temporary pacing for tachyarrhythmia or bradyarrhythmia, may be needed to achieve hemodynamic stabilization. The development of conduction abnormalities is significantly associated with higher mortality ^(53)^. Importantly, atrioventricular block, bundle-branch block, and intraventricular conduction delay may be the first sign of myocarditis ^(24), (38)^; thus, consultation with cardiologists is recommended when these ECG changes are encountered during surveillance for ICI-related CAEs.

### Pericardial diseases

There have been several case reports and case series that described pericardial diseases presenting pericarditis, pericardial effusion, or cardiac tamponade after ICI treatment, but little is known about clinical significance of ICI-related pericardial diseases ^(22)^. In a retrospective study of 3,966 ICI-treated patients, 14 patients (0.35%) required pericardiocentesis, while the incidence of pericardiocentesis was 0.11% in patients receiving non-ICI therapy ^(54)^. This study suggests that ICIs may increase the risk for pericardiocentesis, but the survival after pericardiocentesis was comparable between patients receiving ICIs and those not receiving ICIs ^(54)^. In addition to colchicine and nonsteroidal anti-inflammatory drugs, corticosteroids were administered to reduce pericardial effusion in most patients who were treated with ICI ^(15), (22), (54)^.

### Takotsubo-like cardiomyopathy

Several case reports and case series described Takotsubo-like cardiomyopathy as manifestation of ICI-related cardiotoxicity ^(15), (16), (40), (55)^. In a pooled analysis of 30 patients with ICI-related complications, Takotsubo-like cardiomyopathy was present in 4 patients (14%) ^(53)^. Takotsubo cardiomyopathy is a syndrome of reversible apical myocardial dysfunction triggered by sudden sympathetic hyperactivation upon stress, not by myocardial inflammation ^(56)^. Whether ICI-related Takotsubo-like cardiomyopathy develops due to direct ICI-related effect on the heart or indirect effect leading to sudden sympathetic surges is unclear. Temporarily interrupting ICI therapy for careful observation is desirable. According to a case report, two patients with ICI-related Takotsubo-like cardiomyopathy almost completely recovered after the use of high-dose corticosteroids ^(55)^.

## Conclusion

ICI-related CAEs, such as myocarditis, arrhythmias, pericardial diseases, and Takotsubo-like cardiomyopathy, are rare but potentially life-threatening. ICIs are widely and increasingly used and will be approved for use in a broader range of cancer. Close monitoring and early detection of signs and symptoms of ICI cardiotoxicity are crucial for prompt and accurate diagnosis, and immunosuppressive therapy (high-dose corticosteroids) should be initiated without delay to improve the outcome of patients with ICI-related CAEs. Interdisciplinary collaboration between oncologists and cardiologists will advance our understanding of pathophysiology and further improve clinical approaches and management of ICI-related CAEs.

## Article Information

### Conflicts of Interest

H.A. has received trust research/joint research funding from Ono Pharmaceutical Co., Ltd., and honoraria for lecture from Daiichi Sankyo Co., Ltd., Bayer Yakuhin, Ltd., and Pfizer Japan Inc; I.K. has received honoraria for lecture from Ono Pharmaceutical Co., Ltd.
